# Endolymphatic Hydrops Magnet Resonance Imaging in Ménière’s Disease Patients after Cochlea Implantation

**DOI:** 10.3390/brainsci13060853

**Published:** 2023-05-25

**Authors:** Christoph J. Pfeiffer, Hans-Björn Gehl, Lars-Uwe Scholtz, Peter Goon, Holger Sudhoff, Ingo Todt

**Affiliations:** 1 Department of Otolaryngology, Head and Neck Surgery, Medical School OWL, Bielefeld University, Klinikum Bielefeld Mitte, Teutoburger Str. 50, 33604 Bielefeld, Germany; 2 Department of Radiology, Medical School OWL, Bielefeld University, Klinikum Bielefeld Mitte, Teutoburger Str. 50, 33604 Bielefeld, Germany; 3 Department of Medicine and Otolaryngology, National University of Singapore and National University Health System, Singapore 117549, Singapore

**Keywords:** cochlea implant, endolymphatic hydrops, magnet resonance imaging, morbus Ménière

## Abstract

Introduction: Cochlear implantation in patients with Ménière’s disease (MD) is the treatment of choice in cases of functional deafness. Additional vertigo control is of central importance in this group of patients. Endolymphatic hydrops (ELH) is the pathophysiological correlate of MD and can be evaluated by magnet resonance imaging (MRI). Bilateral MD occurs in 10–33% and can be the reason for a postoperative persisting or newly occurring vertigo in this group. Recent developments in the field of implant magnets and experience in MRI sequences allow the diagnostic performance of MRI in cochlear implantees to be evaluated. The aim of the present study was to evaluate the possibility of MRI as a visual diagnostic tool for endolymphatic hydrops in cochlear implantees. Material and Methods: This was a retrospective study including three cochlear implantees (age: 61–76 years, one female, two male) suffering from MD who, postoperatively, had a recurrence of vertigo with Ménière’s-like symptoms. An MRI was performed for the evaluation of ELH (ELH-MRI). MRI observation was performed by a 4 h iv. delayed Gad 3 D Flair sequence. Results: In all cases, the ipsilateral implant magnet artifact covered the vestibulum, the semicircular canals and the cochlea. The contralateral vestibulum, the semicircular canal and the cochlea were fully observable, and a classification of the ELH-MRI could be performed. Conclusion: ELH-MRI scanning allows for the detection of contralateral labyrinthine endolymphatic hydrops and is a tool for the postoperative evaluation of vertigo in cochlear implantees.

## 1. Introduction

Cochlear implantation (CI) is the treatment of choice for patients with deafness and insufficient speech perception with hearing aids. Besides these groups, subgroups of patients with comorbidities such as anatomical or structural anomalies, vestibular schwannoma and Ménière’s disease (MD) merit specific consideration for a CI.

Patients with MD are particularly challenging because treatment for the hearing deficit and the vertigo needs to be addressed. The natural course of MD includes bilateral disease in 10–33% [1,2,3,4,5]. Therefore, knowledge about the state of the disease even after implantation on both sides is of great importance.

Developments in MRI-based visualization have made it possible to estimate the labyrinthine endolymphatic hydrops (ELH), which is a pathophysiologic correlate of MD [6,7,8,9]. Different MRI sequences have shown a correlation between the depicted grade of hydrops based on the specific cochlear and vestibular radiologic pattern and the clinical symptoms of MD [7,10,11]. Additionally, due to the ELH-MRI-sequence for the patients, the decision for laterality in unclear cases can be made easier [8]

This highly important development in technology has made it essential clinical practice for cochlear implantees who suffer from MD, as those in this group have particularly challenging problems, since vertigo control is, in many cases, the central motivation for these patients due to the combination of hearing loss and vertigo with, in many cases, increasing attack recurrence.

Since the development of a bipolar diametral implant magnet in 2014 (Synchrony Implant, MEDEL, Innsbruck, Austria), many problems with performing MRI in patients with cochlear implants have been addressed. MRI scans are now possible without complications in terms of pain and demagnetization up to 3 T and even without a head wrap [12].

Specific positioning of the implant and the head inside the scanner have made it possible to visualize the cochlea and the internal auditory canal [13,14,15] and allowed for tumor follow-up of the vestibular/intralabyrinthine schwannoma and the estimation of cochlear implant electrode positions [16,17].

The artifact size related to the cochlear implant magnet is the key limiting factor for the clinical application of different sequences [18]. Besides the known increased artifact size of 3D sequences, different publications have focused on the influence of the use of different sequences in general and the application of MARS (magnet artifact reducing sequences) in detail such as MAVRIC and SEMVAC [19,20,21,22].

The effect of ELH specific sequences on the MRI artifact and therefore the visualization of the structures of interest (cochlea, semicircular canals (SCC) and vestibulum) in cochlear implantees is unknown.

Another challenging point is the clinical value and interpretation of the state of an ELH in a cochlear implantee. The occurrence of vertigo after CI is well known and can be caused by different reasons such as vestibular receptor destruction, perilymphatic fistula, compensative problems, costimuation, etc. [23]. Knowledge about the ipsilateral or contralateral ELH would be important additional information in this complex field of postoperative vertigo in cochlear implantees.

The results of previous studies suggest that the depiction of the inner ear, internal auditory canal and cerebellopontine angle by MRI in cochlea implantees depends on the used MRI sequence-related artifact. The aim of the present study was to evaluate the feasibility of assessment for ELH by an MRI on the ipsilateral and contralateral side in cochlear implantees, although there is an artifact.

## 2. Materials and Methods

Patients: In this retrospective study, we examined three cochlear implantees (implantation between 2020 and 2021, patients’ age between 59 and 76 years, two male and one female). Two of them suffered from MD according to Barany society and AAOHNS 2015 [24,25]. In the cases of these two patients, a definite MD was present. According to the criteria of the societies, these patients would suffer from two or more spontaneous episodes of vertigo. The episodes would last 20 min to 12 h each. Furthermore, there would be a documented low-to-medium-frequency sensorineural hearing loss at the index ear at a minimum of one time during or after an incident of vertigo. Moreover, there would be volatile aural symptoms in the form of hearing, tinnitus or fullness in this ear. Additionally, there would be no better explanation by any other vestibular diagnosis [24,25].

One patient showed functional deafness and Ménière-like symptoms. In cases of MD, we regularly perform an MRI to foreclose intra- and retrocochlear disorders and to gauge endolymphatic hydrops. All three patients had a recurrence of vertigo after the initial cochlear implantation. An MRI was performed to evaluate for endolymphatic hydrops. MRI-enabled cochlear implant systems were implanted in all patients, without the need to wrap the head during the MRI examination (2 × MEDEL Synchrony 2, 1 × AB High-Res 3D).

MRI: Scanner and sequence: A 3 tesla MRI system (Ingenia, Philips, Best, The Netherlands) with a 16-channel array head coil was used. Gadobutrol at a dose of 0.2 mL/kg was applied intravenously 4 h before scanning. The imaging protocol consisted of a three-dimensional fluid-attenuated inversion recovery (3D-FLAIR) sequence. The parameters for the 3D-FLAIR sequence were: time of repetition 6000 ms, time of echo 177 ms, time of inversion 2000 ms, matrix size 240,217, field-of-view 190 mm, slice thickness 0.8 mm, acquisition time 12 min.

Graduation: The estimation of ELH grading was described for the vestibular part by using a four-stage graduation system [26] and for the cochlear part by using the four-stage graduation [11,27].

Ethics: The procedures conformed to the World Medical Association’s Declaration of Helsinki and were approved by the University of Münster Faculty of Medicine and Health Sciences Research Ethics Committee (reference: 2022-314-f-S). All participants gave their written informed consent.

## 3. Results

In all three patients, the artifacts being caused by the ELH-MRI sequence meant that it was not possible to visually evaluate the inner ear, the internal auditory canal or the cerebellopontine angle of the implanted side after CI.

However, despite the artifact, the visualization of the inner ear, internal auditory canal and cerebellopontine angle by MRI of the contralateral side was possible. Even the visualization and graduation of an ELH of the non-implanted side was possible by means of the special hydrops sequence.

Patient 1: A 64-year-old man was diagnosed with single-sided definite MD 26 years ago. Between the attacks, he underwent oral therapy with betahistine and an endolymphatic sac surgery 12 years ago. Despite this therapy, there were recurrent attacks of vertigo and hearing loss. In the last few years, there was increasing hearing loss on the side with diagnosed MD (right side). He therefore suffered from functional deafness in this ear and underwent CI, endolymphatic sac surgery revision and occlusion of the lateral SCC [28].

On the left side, there was also moderate hearing loss, especially in the high frequencies with a disturbance up to a 50 dB hearing level between 2 to 6 kHz. The caloric test showed a normal response on the left side but no response on the right side. On both sides, there were no pathological saccades in the video head impulse test.

**Figure 1:**
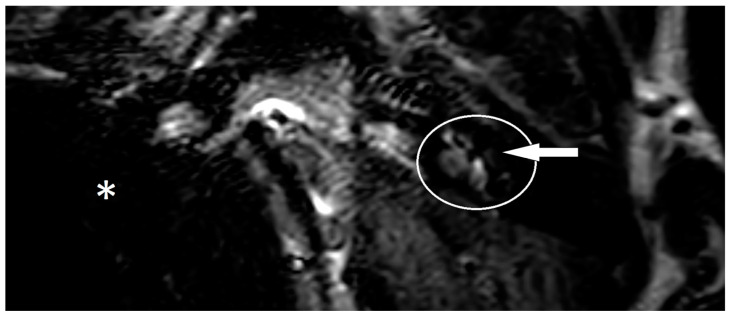
MRI of patient one in a three-dimensional fluid-attenuated inversion recovery (3D-FLAIR) sequence with delayed contrast. The left inner ear is marked with a white circle. The implanted side cannot be seen because of an artifact of the implant. The artifact is marked with a white star. It is possible to mark out a cochlear hydrops grade 1, which is marked with a white arrow.

One year after the CI, the patient suffered from new attacks of rotational vertigo. There was no change in the vestibular examinations. An MRI with an ELH sequence was performed, and a cochlear ELH grade 1 was also observed on the left side (Figure 1). Since that time, the patient has had no further attacks and is stable, so no escalation of therapy was necessary.

Patient 2: A 60-year-old female suffered from rotational vertigo attacks, tinnitus, and increased hearing loss and was diagnosed with definite MD 2 years ago. Functional deafness in that left ear necessitated a CI, endolymphatic sac surgery and an occlusion of the lateral semicircular canal. The right ear showed discreet high-frequency hearing loss of a 30 dB hearing level at 6 kHz. The caloric test showed a normal response on both sides.

One year later, the patient presented with vertigo again, and a recovering of the round window membrane and electrode insertion place was performed to exclude a perilymphatic fistula. The pure tone audiometry and the caloric test showed no change.

**Figure 2:**
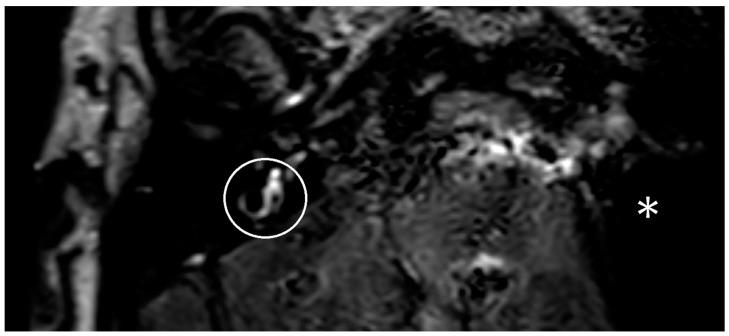
A visualization of a three-dimensional fluid-attenuated inversion recovery (3D-FLAIR) sequence with delayed contrast of the MRI of patient two. The right inner ear is marked with a white circle. Because of an artifact of the implant, the implanted side cannot be seen. The artifact is labeled with a white star. There is no hydrops on the right side.

In the following months, there was increasing hearing loss on the right side. Besides the hearing loss, new caloric testing indicated a conspicuous change on the contralateral side too. In the following months, the patient showed further vertigo attacks and new hearing loss on the contralateral ear. An MRI with a hydrops sequence was performed, but no hydrops was demonstrated on the right side (Figure 2).

The patient underwent gentamicine topical therapy on the cochlear implanted side (left) to control the vertigo. There were no new attacks after that therapy. Further therapy was not required.

Patient 3: A 76-year-old male initially presented with bilateral sensorineural hearing loss at our clinic. There was hearing loss up to a 60 dB hearing level from 0.75 to 4 kHz on the right side. The hearing loss was more severe on the left side. There was only an air conduction threshold, with a decrease from a 70 dB hearing level between 0.25 and 1 kHz to a 95 dB hearing level from 4 to 6 kHz.

At this time, the caloric test showed a regular caloric bilateral response, and there were no pathological saccades in the video head impulse test.

We performed a CI on his left side. Two months after the implantation, the patient suffered from rotational attacks of vertigo with a spontaneous nystagmus to the opposite ear. These attacks were suspicious for MD because of their duration. The caloric test showed no response at the implanted ear at the time. Furthermore, the head impulse test showed a new abnormality in the right horizontal SCC in comparison to the preoperative testing.

**Figure 3:**
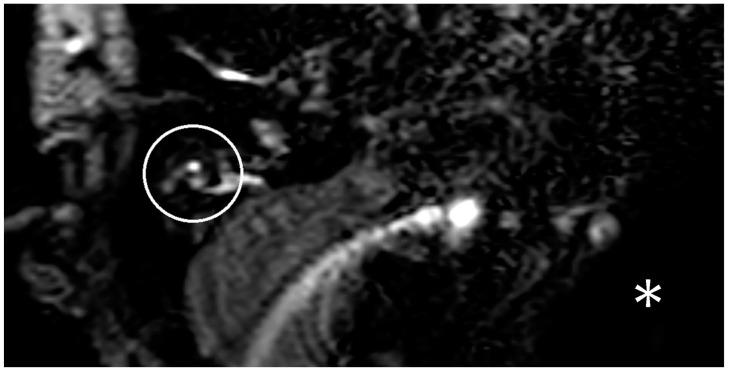
A depiction of a three-dimensional fluid-attenuated inversion recovery (3D-FLAIR) sequence with delayed contrast of the MRI of patient three. The right inner ear is marked with a white circle. There is also an artifact on the implanted left side, which is denoted with a white star. There is no hydrops on the not-implanted side.

It was decided to perform an MRI with a hydrops sequence to exclude an ELH on the unimplanted ear (right side). The MRI with a hydrops sequence, which was performed, presented no hydrops on the right side (Figure 3). The patient was diagnosed with vestibular neuronitis. During the attack, the patient underwent intravenous therapy with prednisolone and dimenhydrinate. There was only one attack, and the patient recovered quickly. There was no need for additional therapy.

## 4. Discussion

Patients with severe sensorineural hearing loss as a sequel of MD who do not benefit from a hearing aid are often managed with CI [29,30]. The control of vertigo often has a greater and more important impact on the quality of life than the improvement in understanding speech [31].

One diagnostic challenge in MD patients who have undergone CI is the frequency of bilateral MD [1,2,3,4]. For example, some patients may develop vertigo postoperatively, and it can be difficult to work this up further with commonly used imaging modalities such as MRI (due to the implanted magnet). In particular, the state of ELH (as the pathophysiological correlate of the MD) is of special interest in these patients.

It is known that ELH can also be present on ears. This can be in cases of the uni- and bilateral occurrence of MD [5,32,33,34].

In the unilateral MD, there can also be an ELH in the unaffected ear [5,32,33]. While the hearing loss is more distinct in the affected ear, the ELH can be similar in both ears [33]. If there is a different degree of ELH in both ears, the more severe hearing loss is in the ear with the higher degree of ELH [32]. If the degree is similar in both ears, the hearing loss is more severe in the affected ear [33].

In cases of Bilateral MD, the degree of the ELH is often larger in the ear that was clinically affected first [5,35,36]. A correlation between the degree of the ELH and the symptoms of MD was discussed [36,37].

Different MRI sequences are known to detect the ELH in patients with MD [26]. It was shown that ELH can be detected after the intravenous injection of Gadobutrol [38,39].

For patients with cochlear implants, the MRI behavior is related to the implant magnet artifact and is of particular interest.

Recent developments in the implant magnet design (e.g., bipolar diametric), the specific positioning of the implant and the head inside the scanner have made the visual assessment of the IAC, cochlea, SCC and vestibulum possible without complications and without the removal of the magnet [12,13,14,15].

Different publications have shown opportunities for limiting the artifact size by specific sequences (MARS) in patients with cochlear implants and other hearing implants [19,20,21,22]. The application of an ELH-MRI sequence is of high clinical value, and the artifact size is so far unknown.

Our results indicate that, using an ELH-MRI sequence, the ipsilateral side after CI is covered by the implant magnet artifact. This holds true for the axial and coronal plane. For the contralateral side, an estimation of the vestibulum, cochlea and SCC was possible. A grading of the ELH for the vestibular system and the cochlea was performed.

We observed one case of cochlear ELH grade 1 and, in two cases, no ELH.

This finding shows that a follow-up of the ELH grade in cochlear implantees with MD is useful and can contribute to the evaluation of causes of vertigo after CI in general and specifically for cochlear implantees with MD.

In this group, it is of high importance to emphasize that causes for the new occurrence of vertigo after CI can be multifactorial. The possibility of a diagnosis of an ELH for this group of patients with MD is of high importance.

One of the limitations of the study is the use of only a single MRI sequence for the estimation of an ELH, which is well established in our radiologic department. We did not use other established sequences [40] and cannot make a statement about the artifact size and the visualization of the ipsilateral and contralateral side after CI for these other sequences.

## 5. Conclusions

ELH MRI allows for the detection of contralateral labyrinthine ELH in cochlear implantees and is a useful diagnostic tool for the postoperative evaluation of vertigo.

## Data Availability

The original data are available from the corresponding author upon request.
